# Supplementary material for: Deep learning model using continuous skin temperature data predicts labor onset
Source: BMC Pregnancy Childbirth. 2024 Nov 25;24:777. doi: 10.1186/s12884-024-06862-9 (PMC11587739; doi:10.1186/s12884-024-06862-9)

Supp Fig 1

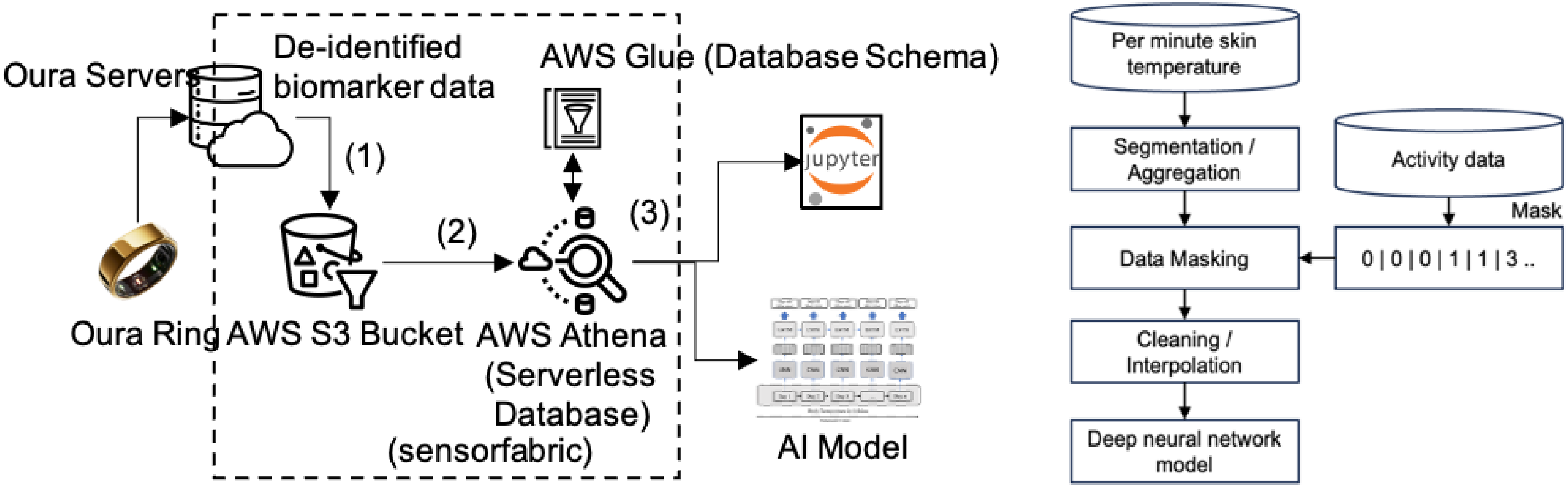

Oura Servers
De-identified
biomarker data
AWS Glue (Database Schema)
(1)
(2)
(3)
jupyter
Oura Ring
AWS S3 Bucket
AWS Athena
(Serverless
Database)
(sensorfabric)
AI Model
Per minute skin
temperature
Segmentation /
Aggregation
Activity data
Mask
Data Masking
0 | 0 | 0 | 1 | 1 | 3 ..
Cleaning /
Interpolation
Deep neural network
model

Supp Fig 2

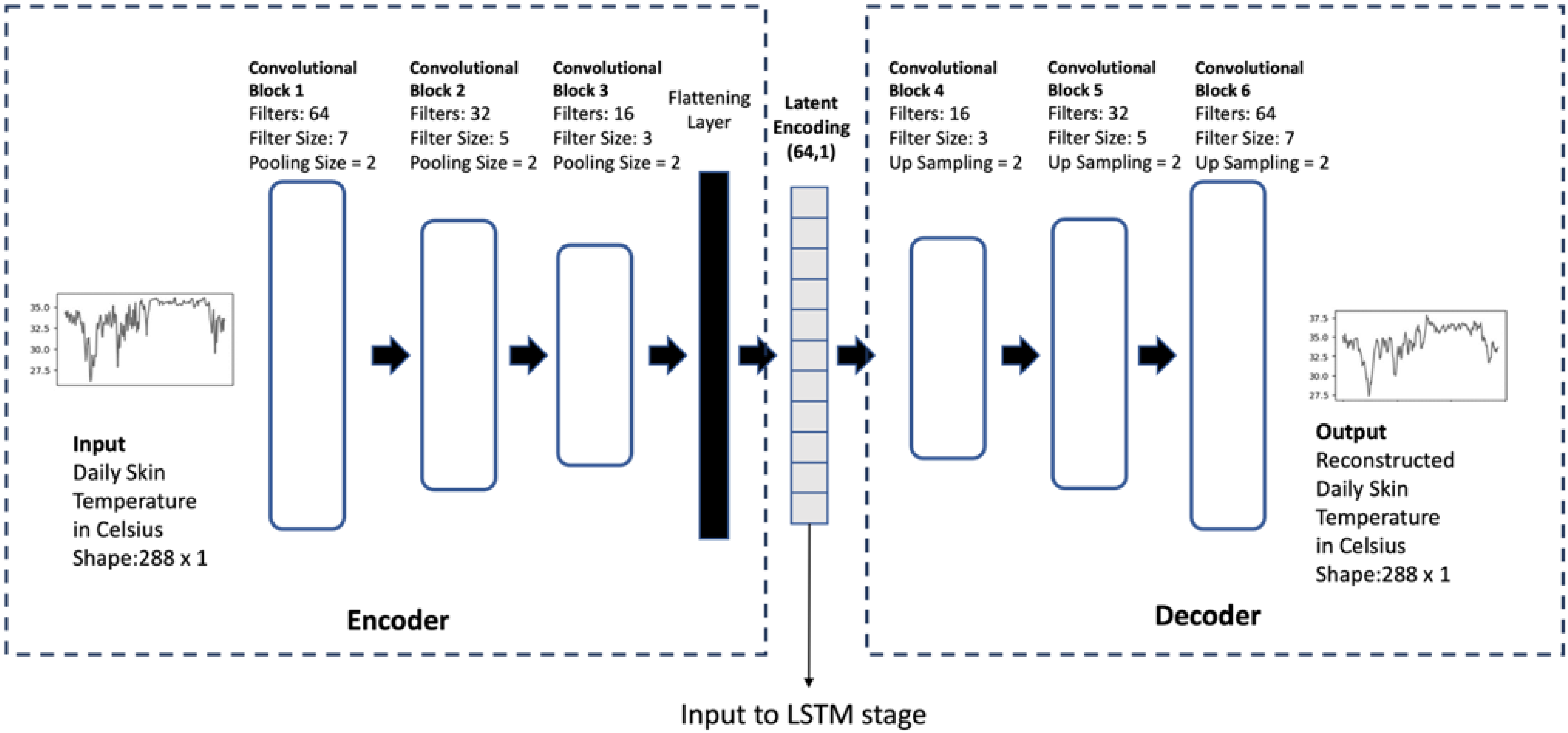

Convolutional Block 1
Filters: 64
Filter Size: 7
Pooling Size = 2
Convolutional Block 2
Filters: 32
Filter Size: 5
Pooling Size = 2
Convolutional Block 3
Filters: 16
Filter Size: 3
Pooling Size = 2
Flattening Layer
Latent Encoding (64,1)
Convolutional Block 4
Filters: 16
Filter Size: 3
Up Sampling = 2
Convolutional Block 5
Filters: 32
Filter Size: 5
Up Sampling = 2
Convolutional Block 6
Filters: 64
Filter Size: 7
Up Sampling = 2
Input
Daily Skin Temperature in Celsius
Shape:288 x 1
Output
Reconstructed Daily Skin Temperature in Celsius
Shape:288 x 1
Encoder
Decoder
Input to LSTM stage

Supp Fig 3

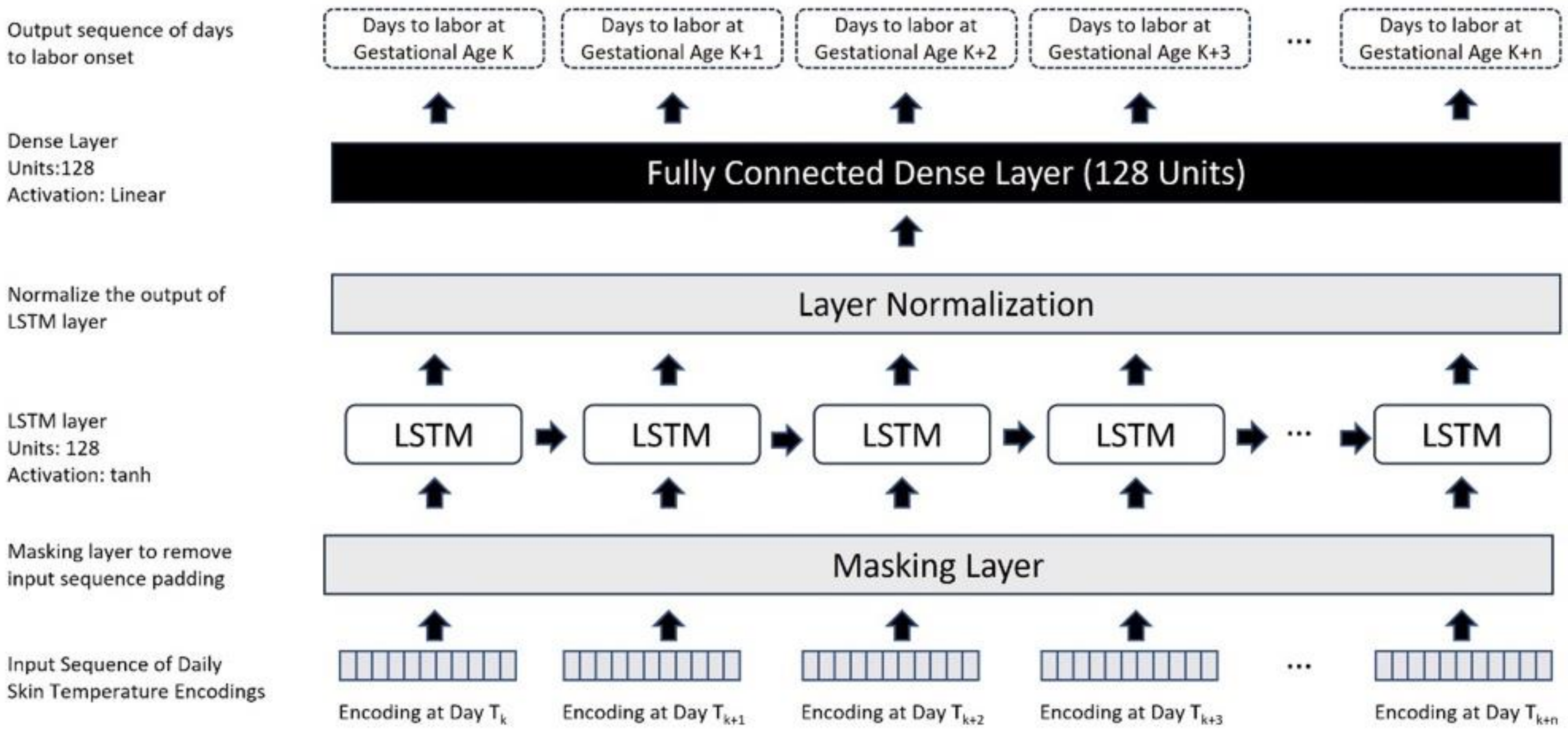
Output sequence of days to labor onset
Days to labor at Gestational Age K
Days to labor at Gestational Age K+1
Days to labor at Gestational Age K+2
Days to labor at Gestational Age K+3
...
Days to labor at Gestational Age K+n
Dense Layer
Units:128
Activation: Linear
Fully Connected Dense Layer (128 Units)
Normalize the output of LSTM layer
Layer Normalization
LSTM layer
Units: 128
Activation: tanh
LSTM
LSTM
LSTM
LSTM
...
LSTM
Masking layer to remove input sequence padding
Masking Layer
Input Sequence of Daily Skin Temperature Encodings
Encoding at Day T_k
Encoding at Day T_{k+1}
Encoding at Day T_{k+2}
Encoding at Day T_{k+3}
...
Encoding at Day T_{k+n}

Supp Fig 4

Spontaneous Labors' Progesterone and Estrogen Metabolites Decrease in the 10 Days Prior to Labor Onset

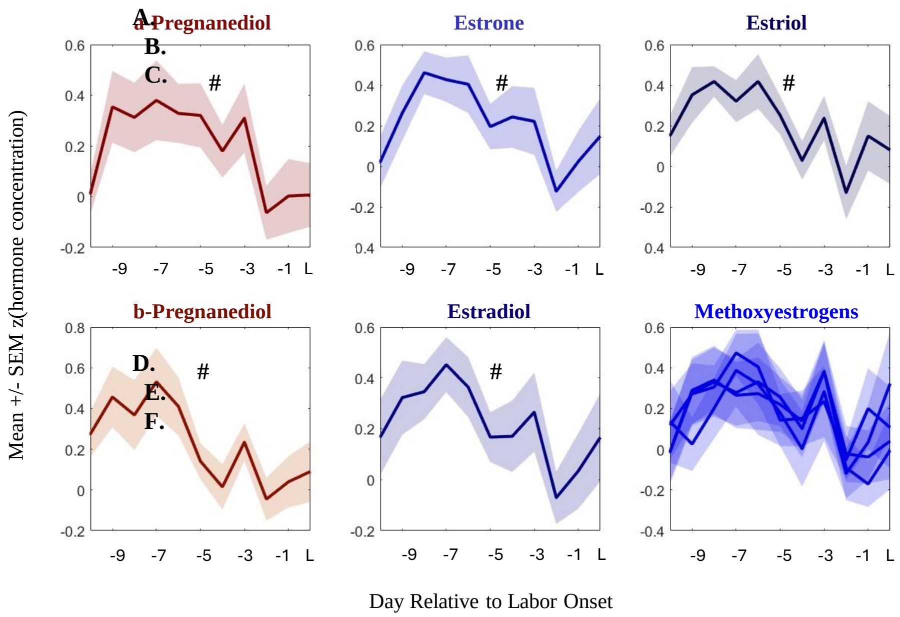

Supp Fig 5

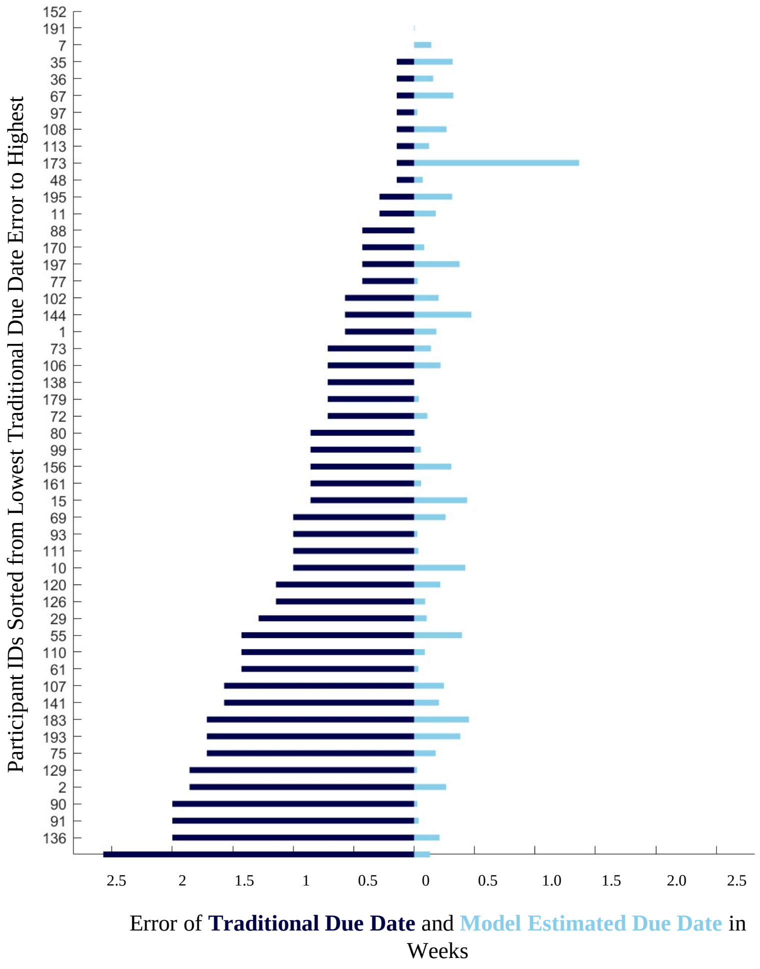
Participant IDs Sorted from Lowest Traditional Due Date Error to Highest
152
191
7
35
36
67
97
108
113
173
48
195
11
88
170
197
77
102
144
1
73
106
138
179
72
80
99
156
161
15
69
93
111
10
120
126
29
55
110
61
107
141
183
193
75
129
2
90
91
136
2.5
2
1.5
1
0.5
0
0.5
1.0
1.5
2.0
2.5
Error of Traditional Due Date and Model Estimated Due Date in Weeks

Supp Fig 6

Spontaneous Individual Z-Scored Temperature Trajectories in the Last Two Weeks of Pregnancy

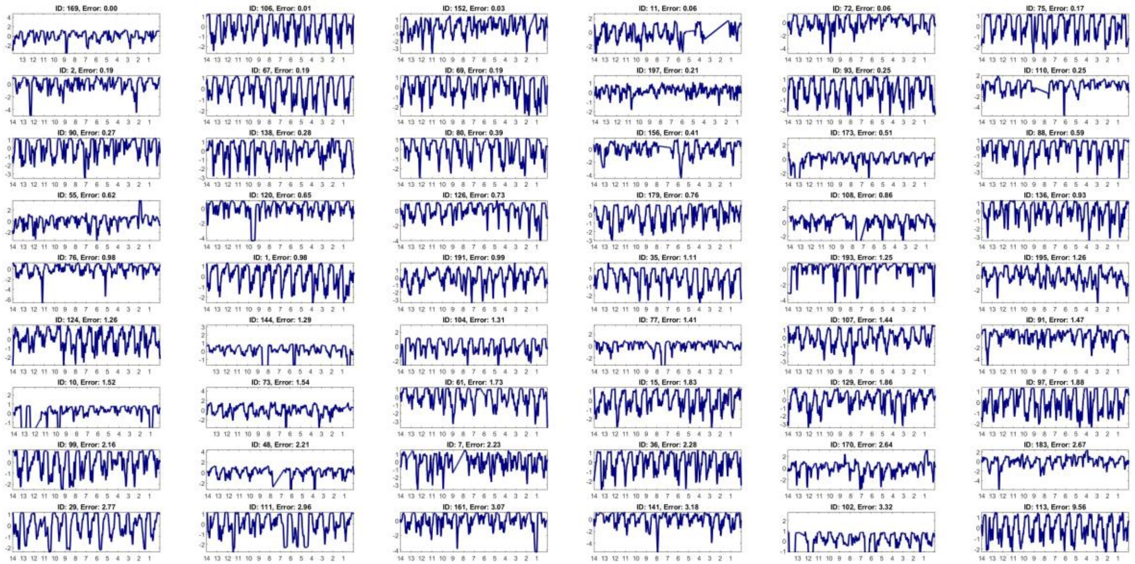

Supp Fig 7

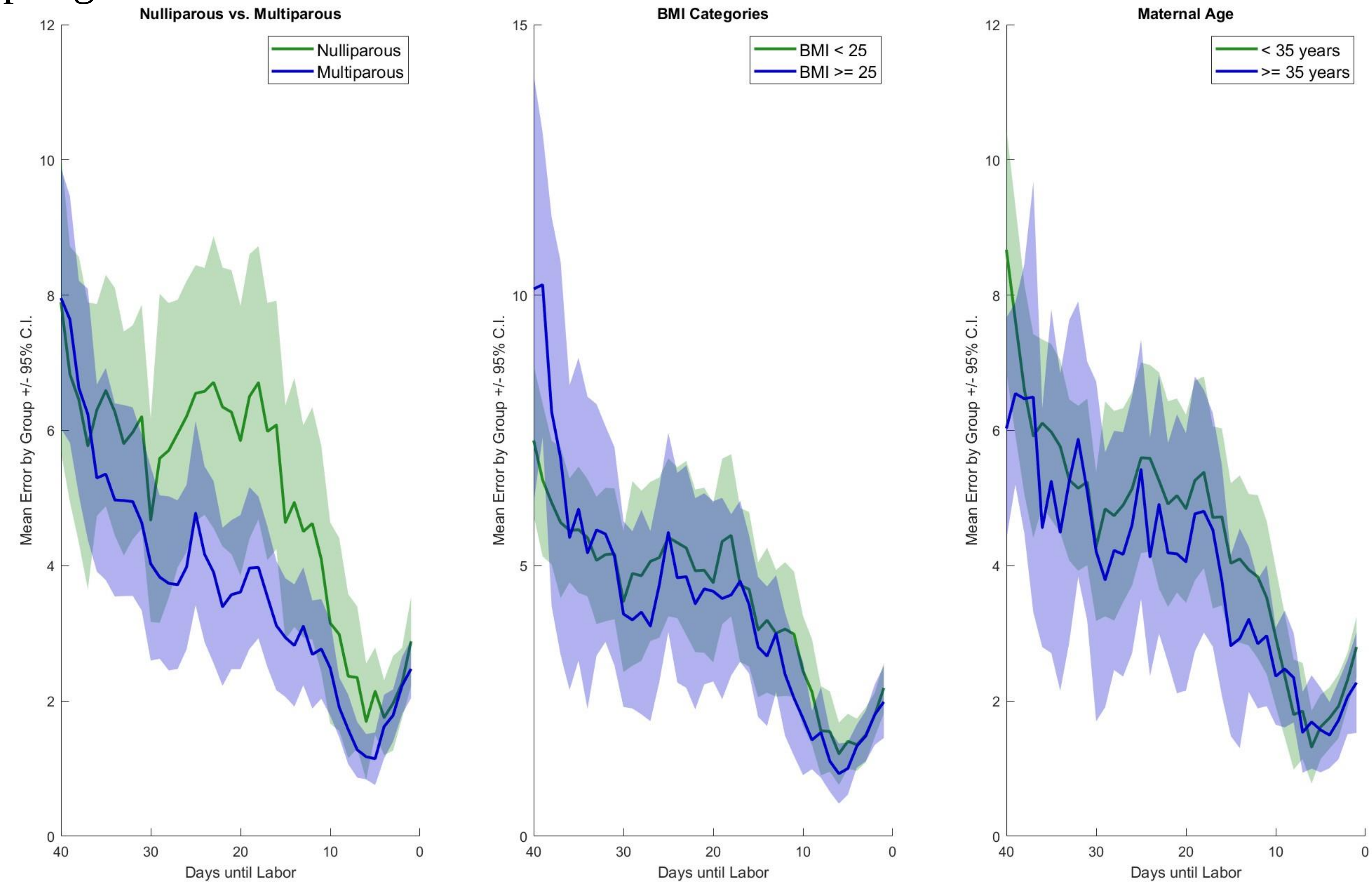

Nulliparous vs. Multiparous
Nulliparous
Multiparous
Mean Error by Group +/- 95% C.I.
Days until Labor
BMI Categories
BMI < 25
BMI >= 25
Mean Error by Group +/- 95% C.I.
Days until Labor
Maternal Age
< 35 years
>= 35 years
Mean Error by Group +/- 95% C.I.
Days until Labor

Supp Fig 8

Model Performance Decays When Data Are Replaced With Zeros, Gaussian Noise, or Adding Noise to Data

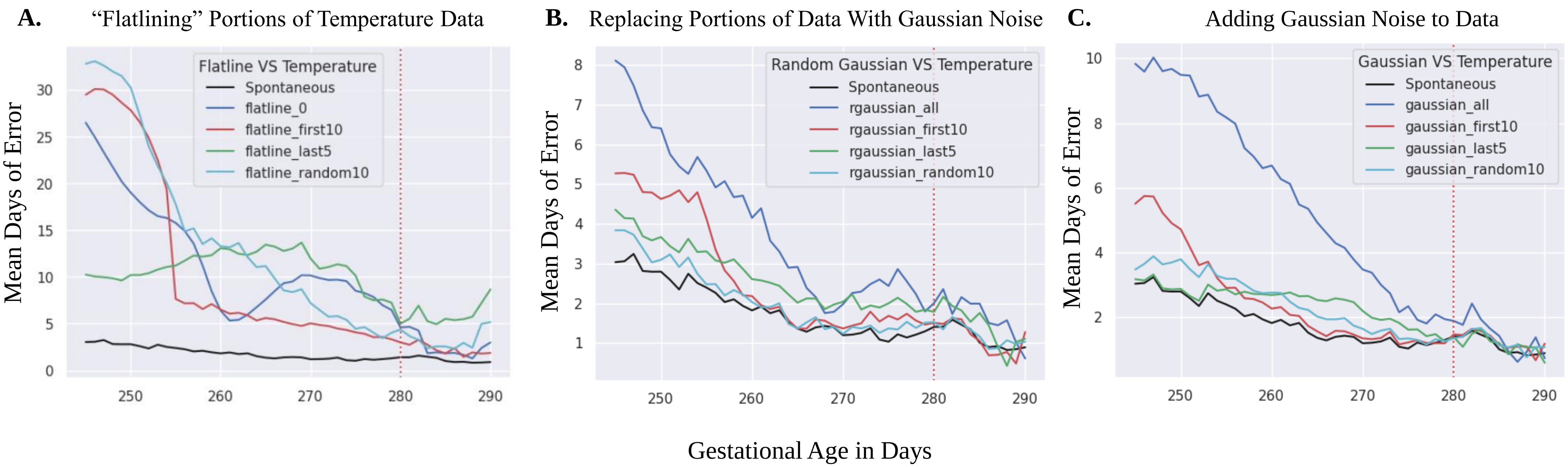

Supplement: Supplementary file 1 — Supplementary Material 1: Supplemental Figure 1. Data Preprocessing and Cleaning. Pre-Processing and Cleaning: data ingestion (A) and pre-processing pipelines (B). A): De-identified biomarker data from Ouraring, including high fidelity temperature and IBI are ingested into a campus secure Amazon Web Services (AWS) S3 bucket indicated by (1). Data is then parsed to generate structured schema, table meta-data in AWS glue, and participant partitions (to accelerate querying of per minute temperature data). These are then fed into a serverless querying solution provided by AWS Athena as shown by (2). B) We pass the raw minute temperatures from the device through pre-processing steps. First, we average the raw values over a 5-minute window, segment it into 24-hour periods starting at 10am each day, which allows the neural network model to learn from daily patterns (both day and night variation). Next, we remove data collected by the ring during non-wear time by using the 5 min activity labels provided by the ring, indicating wear/non-wear. Finally, we employed linear interpolation to account for missing and non-wear daily data. The final output is fed to a DNN model. Supplemental Figure 2. Autoencoder Structure. Supplemental Figure 2. Auto encoders are divided into 2 parts – encoder and decoder. The encoder is responsible for converting values from feature space to latent space, while the decoder is responsible for converting them back to the feature space. AE train in an unsupervised setting where the object loss function MAE, measures the loss of reconstructing the original signal from the latent representation. In the encoder part of the AE, Input data of size 288 is fed into a series of three convolutional blocks. Each convolutional block comprises of a 1-D convolutional layer coupled with a max-pooling layer that enables reduction in data dimensionality. Output from the final convolutional layer is flattened and fed into a dense fully connected layer to produce the e [file 12884_2024_6862_MOESM1_ESM.pdf]
